# The Role of Oxidative Stress and Antioxidant Balance in Pregnancy

**DOI:** 10.1155/2021/9962860

**Published:** 2021-09-27

**Authors:** Tarique Hussain, Ghulam Murtaza, Elsayed Metwally, Dildar Hussain Kalhoro, Muhammad Saleem Kalhoro, Baban Ali Rahu, Raja Ghazanfar Ali Sahito, Yulong Yin, Huansheng Yang, Muhammad Ismail Chughtai, Bie Tan

**Affiliations:** ^1^College of Animal Science and Technology, Hunan Agricultural University, Changsha, 410128 Hunan, China; ^2^Animal Science Division, Nuclear Institute for Agriculture and Biology College, Pakistan Institute of Engineering and Applied Sciences (NIAB-C, PIEAS), Faisalabad 38000, Pakistan; ^3^Department of Animal Reproduction, Faculty of Animal Husbandry and Veterinary Sciences, Sindh Agriculture University, Tandojam, Sindh 70050, Pakistan; ^4^Department of Cytology & Histology, Faculty of Veterinary Medicine, Suez Canal University, Ismailia, Egypt; ^5^Department of Veterinary Microbiology, Faculty of Animal Husbandry and Veterinary Sciences, Sindh Agriculture University, Tandojam, Sindh 70050, Pakistan; ^6^Department of Animal Products Technology, Faculty of Animal Husbandry and Veterinary Sciences, Sindh Agriculture University, Tandojam, Sindh 70050, Pakistan; ^7^Institute of Neurophysiology, University of Cologne, Cologne 50931, Germany; ^8^Institute of Subtropical Agriculture, Chinese Academy of Sciences, Changsha, 410125 Hunan, China; ^9^Hunan International Joint Laboratory of Animal Intestinal Ecology and Health, Laboratory of Animal Nutrition and Human Health, College of Life Sciences, Hunan Normal University, Changsha, Hunan 410081, China

## Abstract

It has been widely known that oxidative stress disrupts the balance between reactive oxygen species (ROS) and the antioxidant system in the body. During pregnancy, the physiological generation of ROS is involved in a variety of developmental processes ranging from oocyte maturation to luteolysis and embryo implantation. While abnormal overproduction of ROS disrupts these processes resulting in reproductive failure. In addition, excessive oxidative stress impairs maternal and placental functions and eventually results in fetal loss, IUGR, and gestational diabetes mellitus. Although some oxidative stress is inevitable during pregnancy, a balancing act between oxidant and antioxidant production is necessary at different stages of the pregnancy. The review aims to highlight the importance of maintaining oxidative and antioxidant balance throughout pregnancy. Furthermore, we highlight the role of oxidative stress in pregnancy-related diseases.

## 1. Introduction

Several reproductive problems have been linked to oxidative stress. Oxidative stress occurs when the body's antioxidant system is depleted owing to an excess of reactive oxygen species (ROS). ROS are highly reactive molecules that are unstable and short-lived. These molecules contribute to the control of signaling pathways, as well as cellular and physiological processes [1]. However, excess ROS may cause cellular toxicity [2]. Animals have an enzymatic antioxidant defense mechanism that suppresses the formation of reactive oxygen species (ROS). It is worth noting that cellular integrity is maintained by a balance of enzymatic and non-enzymatic antioxidant systems. When oxidative stress increases, both antioxidant systems are depleted resulting in reproductive problems [3, 4]. Enzymatic antioxidants like glutathione peroxidase (GPx) and superoxide dismutase (SOD) are antioxidants to neutralize free radicals. On other hand, the non-enzymatic antioxidants such as vitamin C, vitamin E, plant polyphenol, carotenoids, and glutathione interrupt free radical chain reactions. Importantly, antioxidants may have therapeutic promise in the treatment of reproductive-related problems [5].

ROS has a biological effect on various reproductive processes, such as oocyte maturation, fertilization, embryo development, pregnancy, as well as oocyte maturation and fertility. A number of research studies, including animals and humans, showed that ROS has been implicated with female reproduction, particularly ovaries [6–8], fallopian tubes [9] and embryos [10].

The primary function of the placenta is to exchange nutrients and oxygen between mother and fetus. Therefore, interference in these functions leads to hypoxia due to oxidative stress. The disruption in placental function is due to many factors resulting in pregnancy complications [11]. A large number of studies reported that pregnancy problems have been associated with overwhelming oxidative stress from the placenta and or maternal tissues [12]. Other mechanisms are also implicated in the etiology of these complications; oxidative stress has evolved to regulate the cellular and molecular pathways such as altered angiogenesis and inflammation to mediate disease outcomes [13]. The oxidative scenario develops due to increased ROS and depletion of the antioxidant system [14]. Though the development of abnormal oxidative stress leads to spontaneous abortion, idiopathic recurrent pregnancy loss and embryogenesis defect [2, 15–18].

Oxidative stress has been linked to a number of metabolic processes that affect animal health and performance [19]. The study of oxidative stress has increased as a result of its role in adverse pregnancy outcomes. Oxidative stress exhibits dual functions, it aids in the maintenance of redox balance and it plays a part in female reproductive processes. As a result, oxidative stress may aggravate IUGR, endometriosis, and other reproductive issues. Oxidative stress also regulates signaling networks including Kelch-like ECH-associated protein 1, Nuclear factor erythroid 2-related factor 2 (Keap1-Nrf2), nuclear factor kappa-B (NF-*κ*B), forkhead transcription factors of the O class (FOXO) and Mitogen-activated protein kinase (MAPK). Lastly, targeting these pathways appears attractive as a potential therapeutic strategy against pregnancy-related anomalies [5].

## 2. Oxidative Stress and Its Regulatory Mechanism

ROS are oxidative metabolic byproducts that play an important part in cellular activity. They are also implicated in a number of pathological diseases, including *in-vitro* and *in-vivo* pregnancy difficulties [20–25]. The factors responsible for overproduction of ROS are ultraviolet radiation, cigarette smoking, alcohol, non-steroidal anti-inflammatory medication, ischemia-reperfusion injury, chronic infections, and inflammatory diseases [26, 27]. The enzyme superoxide dismutase converts the superoxide anion radical to hydrogen peroxide and oxygen [28], and catalase eliminates hydrogen peroxide when its quantities in the cell are higher [29]. Glutathione reductase is found throughout the body tissues and operates similarly to GPx. Using several systems; the GSR enzyme reduced oxidized glutathione by utilizing NADPH [30, 31].

The secondary defense is based on the GPx enzyme, which possesses peroxidase activity and may eliminate lipid hydroperoxides irrespective phospholipase A2 [32]. There are also a number of oxido-reductases that catalyse thiol and other protein reduction processes. Protective enzymes against free radicals are produced once the cellular components have been oxidatively damaged. For example, DNA nuclear enzymes are known to protect DNA from oxidative damage induced by free radicals [33]. Vitamin E functions as a cofactor for glutathione peroxidase enzymes, and its presence in all cellular membranes suggests that it can protect lipids from oxidation. The ascorbic acid-GSH redox couple directly reduces the tocopherol radical. While *β*-carotene functions in concert with vitamin E, which is a strong scavenger of free radicals, but *β* -carotene only works at low oxygen pressure. Vitamin E, on the other hand, protects *β*-carotene against oxidative damage [34]. In addition, some antioxidants work as free radical quenchers [35]. Early pregnancy deficiency in antioxidants has been associated with the development of maternal-related disorders such as gestational hypertension, gestational diabetes, and other complications [36]. Therefore, the generation of ROS molecules controls several signaling pathways that govern a variety of cellular functions. The activation of these signals causes a change in cellular function, which has a pathogenic effect on the cell [37].

## 3. Oxidative Stress Scenarios in Pregnancy

In normal pregnancy, the developing tissues and organs of the fetus require enough nutrition and oxygen. These processes generate ROS in both maternal and fetal tissues that influence fetal growth development. To provide a suitable environment for the fetus and maternal body, the balance between ROS and antioxidants could be maintained [38]. During pregnancy, the body undergoes numerous physiological changes. The evidence of ROS formation in the second trimester of pregnancy was assumed by the researchers. Increased production of ROS occurs due to the enhanced metabolism, high consumption of oxygen and utilization of fatty acids. During third trimester of pregnancy, increase insulin resistance, fat catabolism, and release of free fatty acids resulting in enhanced production of hydrogen peroxide [39]. Placental cells have a lot of mitochondria, which are the main source of pro-oxygenates. The superoxide anion radical produces more radical species and their generation rises as the pregnancy continues.

Several studies have found that oxidative stress is linked to pregnancy complications that may influence fetal development. The major causes are a lack of nutrition and oxygen for developing fetuses, which causes hypoplasia and disrupts placental function [39, 40]. The difference in total plasma antioxidants status between pregnant and non-pregnant individuals has been observed, implying a low level in the first phase of pregnancy. The total antioxidant capacity of a pregnant woman increases during the second and third trimesters, and by the last week of pregnancy, it has reached the level of a non-pregnant woman. TAC activity increases after the 8^th^ week of pregnancy, and these changes are linked to differences in plasma uric acid levels [41]. Furthermore, reduced TAC levels in pregnancy have been linked to low levels of serum albumin, bilirubin, and vitamin E [42]. As result, it appears that plasma SOD activity is reduced during pregnancy [43]. The SOD reduction promoted triglycerides, total cholesterol, and low-density lipoprotein (LDL) cholesterol levels in blood plasma. Therefore, SOD refers as indicator of oxidative stress and lipid peroxide activity followed by 25 weeks of pregnancy. As a result, lipid peroxidation levels in the blood are higher in pregnant women, serving as a marker of oxidative stress. Previous studies have found that supplementing pregnant individuals with the dietary vitamins, antioxidants, and minerals enhanced TAC activity [42–44].

## 4. Oxidative Stress in Ovary, Uterus and Placenta

Almost every stage of pregnancy is affected by ROS. ROS is known to be the important regulator of ovarian cellular activity [45]. The ROS positive impact has been already mentioned. Previous studies showed that the presence of SOD in ovary, copper-zinc SOD (Cu-Zn SOD) in granulosa cells of follicles and manganese superoxide dismutase (MnSOD) in luteal cells of the corpus luteum in rats [46]. The sources of ROS in the follicles are macrophages, leukocytes and cytokines [26]. Ovulation is dependent on concentration of ROS. ROS suppressors have been demonstrated to interfere with the ovulatory process [47]. Follicles development is associated with an increased metabolic function of granulosa cells, particularly excess amount of cytochrome P450 and steroidogenesis [48]. The presence of ROS in pre-ovulatory follicles alters blood flow and finally leads to follicle rupture [49]. Furthermore, FSH stimulates the synthesis of estrogen, while the overexpression of CAT in developing follicles protects them from apoptosis, ensuring that ovarian function is preserved [50]. Depletion of oxygen is required for follicular angiogenesis [6]. The corpus luteum contributes to functional luteolysis by producing ROS. During the luteal phase, both the ROS and antioxidants are linked to progesterone production [51]. The beneficial effects of ROS and antioxidants in female reproductive and pregnancy outcomes are depicted in Table 1.

The developing fetus has a high energy requirement due to the placental hyperactive metabolic rate, resulting in oxidative stress [52]. Of note, that superoxide anions produced by placental mitochondria appear to be the essential source of ROS and lipid peroxidation in the placenta [53]. As the pregnancy progresses, mitochondrial synthesis of lipid peroxides, free radicals, and vitamin E may also increase [54]. The placenta and large blood arteries mature slowly in the second phase of the pregnancy. After that, maternal blood pumps via interstitial space into the mother's spiral artery [54, 55]. Free radicals are abundant in placental tissues, and oxidation occurs throughout the process. With the help of antioxidant activity, the placenta can slowly adapt to the environment after recovering from stress [40].

SOD activity decreases during the late luteal phase due to increased amounts of lipid peroxide. Importantly, ROS are known to have a role in numerous phases of the endometrial cycle, and may also produce PGF_2_ through NF-*κ*B activation [56]. Estrogen and progesterone levels dropped significantly as a result of lower SOD expression. In a consequence, ROS accumulates in the uterus, leading to implantation failure. The basal level of ROS controls angiogenic activity in the endometrium and results in endometrial regeneration during each cycle. Thus, appropriate ROS concentration is critical for normal homeostasis. However, an increased level of ROS from the placenta has been associated with pregnancy-related disorders [57–59]. The TNF-*α* cytokine that influences endothelial cell dysfunction and the antioxidant Mn-SOD are both disrupted and have protective effects. The production of cytokines and prostaglandins is increased by ROS-related poor placental function, producing endothelial cell injury and contributing to preeclampsia [60].

## 5. Regulation of Multiple Signaling Pathways by Oxidative Stress

Oxidative stress has been linked to influence signaling pathways, particularly in reproductive diseases ranging from egg production to ovulation. It alters immune system of the uterus resulting in embryonic failure [61, 62]. Oxidative stress has also been involved in regulating molecular pathways in reproductive disorders such as p38 MAPK, Keap1-Nrf2, the Jun N-terminal kinase (JNK), the FOXO family, and apoptotic pathways. Therefore, the research on this aspect may yield new insights that might influence female reproductive system.

Nrf2 is a signaling molecule that protects cellular function by acting as an antioxidant in response to oxidative stress [63]. Physiologically, Nrf2 binds with Keap1 in the cytoplasm before being degraded by the proteasome [64]. Once the Nrf2 is activated, it translocate into nucleus, where it activates several antioxidant genes [65]. In contrast, activation of antioxidant genes and restoration of vascular redox homeostasis are required when OS is evident suggesting the crucial function of Nrf2 [66]. The deficiency of Nrf2 induced fetal DNA damage and neurological discrepancies and inactivation of Nrf2 were also exhibited inflammation triggered trophoblastic apoptosis. Previous evidence showed that Nrf2 plays an important role in pregnancy and protects the fetus from OS *in-utero* [67]. The maternal immune system is susceptible to Nrf2. Nrf2 is only decreased once the full-term foetus is delivered in a normal pregnancy. When a fetus is infected *in utero*, the Nrf2 expression is favorably reduced [68]. In the case of OS-induced metritis, it is expected that Nrf2 would be considerably decreased, and Keap1 would bind to Nrf2. Similarly, FOXO3 is essential in the interaction between Keap1 and Nrf2. In the absence of FOXO3, Nrf2 is activated by AKT and protects cells against OS [69]. Lastly, we hypothesized that OS causes inflammation in the reproductive system, with FOXO3 playing a role in the interaction between Keap1 and Nrf2, which may be used as a marker for OS insults.

NF-*κ*B is an inert molecule, its family comprises five transcription factors c-Rel, p50, p52, RelB and RelA (p65) [70]. NF-B is a redox-sensitive transcription factor that is the primary regulator of the inflammatory response [71]. Therefore, the beneficial effects of NF-*κ*B are evident in embryonic stress that activates NF-*κ*B and other diverse inflammatory cytokines which persuades apoptosis within placenta [72]. Hence, it was concluded that NF-*κ*B plays an important role in the cell survival by releasing anti-apoptotic genes. In normal conditions, NF-*κ*B is bound to inhibitory I*κ*B proteins and remains inactive in the cytoplasm. The breakdown of I*κ*B proteins activates NF-B, which subsequently translocate into the nucleus and generates desirable genes, whereas I*κ*B proteins are mediated by the I*κ*B kinase (IKK) complex (IKK*α* and IKK*β*) [73]. Increased expression of NF-*κ*B in cultured endometrial stromal cells has been found in reproductive diseases such as endometriosis [74]. Altered production of NF-*κ*B production has been associated with inflammation. Endometriosis is a condition induced by OS which increases the concentration of TNF-*α*, resulting in inflammation thereby; NF-*κ*B is activated. Moreover, IL-1*β* activates NF-*κ*B, which in turn produces inflammatory cytokines [75], comprising macrophage migration inhibitory factor (MIF) in endometrial stromal cells [76] and TNF-*α* in immortalized epithelial (12Z) cell line [77]. In summary, OS-mediated reproductive disorders are caused by NF-*κ*B activation.

FOXO1 and FOXO3 have been contributed to OS and pregnancy. The FOXO subfamily of Forkhead transcription factors is a direct downstream target of the PI3K/Akt pathway [78]. The family of FOXO proteins is involved in different biological processes such as proliferation, apoptosis, autophagy, metabolism, inflammation, differentiation and stress tolerance [79]. The FOXC1 displays a pivotal role in reproduction and also mediates cyclic differentiation and apoptosis in normal endometrium [80]. Recent studies have shown that FOXO1 knockdown disrupts the expression of over 500 genes in decidualized human endometrial stromal cells [81]. Previous research has shown that FOXO transcription factors can control multiple gene responses to change hormone levels [82]. Besides, that FOXO1 is also responsible for the induction of decidual marker genes, including WNT4, prolactin (PRL) and insulin-like growth factor-binding protein 1 (IGFBP1) [83].

Three signaling molecules are triggered by the extracellular milieu, including ERK, which is activated by inflammation and growth factors, and JNK and p38 MAPK, which are mostly activated by stress and inflammation [84]. It has been shown that ERK activation is increased in endometrial tissue, suggesting that ERK may play a role in endometriosis and phosphorylated ERK is increased in primary eutopic epithelial cells [85]. ERK activation can also be influenced by oxidative stress. In response to normal women, hydrogen peroxide causes ERK phosphorylation in endometriotic stromal cells [86].

## 6. Contribution of Oxidative Stress in Pregnancy Complications

### 6.1. Intrauterine Growth Restriction

Intrauterine growth restriction (IUGR) is a pregnancy ailment in which an underweight/incomplete fetus develops in the uterus [87]. The causes are multifactorial such as maternal, fetal, placental, infectious, or genetics [88]. About 76% of intrauterine deaths have been associated with IUGR [89]. The most significant cause of IUGR is utero-placental dysfunction occurs due to the congested maternal utero-placental blood flow [90]. Proper functioning of the placenta requires greater energy demand for cell growth, proliferation and metabolic activity which in turn produce oxidative stress. Oxidative stress plays an essential role against various stimuli which influence placental function [91]. Cellular injury occurs as a result of lipid peroxidation and fatty acid oxidation, and it is mostly utilised to identify oxidative stress indicators [92]. Evidence of IUGR in livestock has been raised through environmental factors and affects goats, sheep, pigs and other animals. Of note, that significant evidence of IUGR exists in multi-fetal animals including pigs. It has been documented those animals with this condition have reduced birth weight, postnatal growth, development and liver dysfunction [93]. A detailed description of IUGR occurrences in clinical and health deviations is well been ascribed in the previous studies [94–96]. More evidence is required to be revealed the underlying molecular mechanisms.

### 6.2. Spontaneous Miscarriage and Recurrent Pregnancy Loss

Spontaneous abortion can be classified as loss of pregnancy before 20 weeks of gestation. The incidence may range from 8-20% in pregnancies and is due to chromosomal aberration, which accounts for 50% of all miscarriages. While, the rest are associated with congenital and uterine malfunctions, infections, maternal diseases and unknown causes [97].

**Figure 1:**
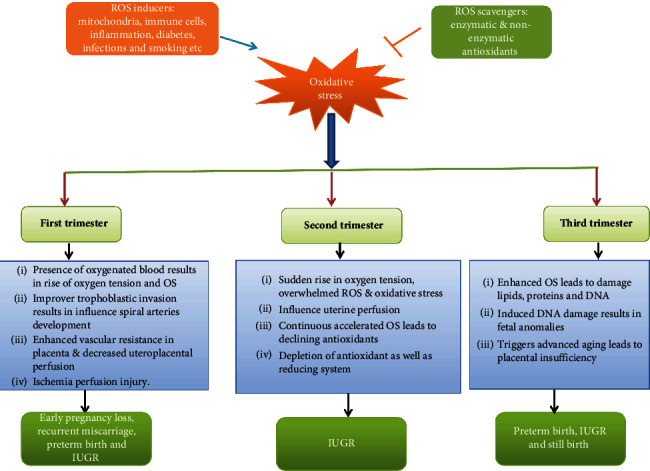
The Impact of Oxidative Stress on Pregnancy Outcomes.

In early pregnancy losses, elevated levels of MDA and lipid peroxides were observed in placental tissues in comparison with controls. Previous studies have shown that overloading of ROS could lead to the premature and sudden formation of maternal placental perfusion [2]. Other evidence reported that oxidative stress damage the trophoblast and ultimately leading to early pregnancy losses. The incidence of oxidative stress occurred due to the depletion of the antioxidants system and thus unable to scavenge free radicals [87, 98]. Although there is diversity in previous studies, it seems to be a relationship between ROS and antioxidants in miscarriage. The abnormal placentation may arise from syncytiotrophoblasts and may be vulnerable to idiopathic recurrent pregnancy loss [97]. Oxidative stress enables the potential to influence pregnancies due to the depletion of antioxidant capacity within the body [99]. The influence of oxidative stress in pregnancy problems is depicted in Figure 1. The issue of recurrent pregnancy losses, research gaps, and their treatment has been thoroughly reviewed [100, 101].

### 6.3. Gestational Diabetes Mellitus (GDM)

GDM is a type of diabetes mellitus in which pregnant women develops glucose intolerance to a different degree [102]. It was reported in 2-5% of pregnancies while; data suggested the incidences increased up to 18% in all pregnancies [103]. GDM develops during the second trimester of pregnancy, causing fetal macrosomia, perinatal mortality, and making mother vulnerable for T2DM [102, 104].

The pregnancy has been linked to an imbalance of pro and anti-inflammatory mediators [105]. The levels of T cells subsets were increased in women with GDM compared to control healthy subjects whereas; T cells expressing CTLA-4, a downregulation of the immune system which lightly expressed in Tregs were suppressed [106]. Changes in the Treg population suggest that the Treg pool in GDM is becoming less active [76]. Thus, it suggests that the lack of immune down-regulation helps maternal-fetal tolerance. Although, the toll-like receptors TLR-2 and TLR-4 stimulate inflammatory cytokines which were enhanced in peripheral blood mononuclear cells of women with GDM [107]. Previous literature revealed the ambiguous results of TNF-*α* in GDM condition [79, 82], but more descriptive role of GDM is well-highlighted somewhere else [108]. An evidence of oxidative stress-related problems during pregnancy is well-reviewed by others [12, 109].

## 7. Antioxidant Approaches in Pregnancy

The detrimental effects of oxidative stress and ROS on female reproduction system have been well illustrated for since long [110]. It was suggested that the generation of ROS is impaired by cytochrome P450 and corpus luteum, which itself is considered a key source. The initiation of oocyte maturation and others processes are mostly affected by different levels of ROS and antioxidants [6]. Endometriosis and unexplained infertility conditions are also linked to the OS [111].

Antioxidant supplementation possess positive effects through a variety of pathways, including direct scavenging of reactive oxygen species (ROS) and damage repair [112]. The protective effects on fertility consisting enhanced blood circulation in endometrium, reduced hyperandrogenism, lowered insulin resistance, and positive impact on prostaglandin synthesis and steroidogenesis [112–114]. A current systematic review indicates the positive impact of antioxidants in female fertility [115]. Antioxidants were also involved in enhancing live birth weight and clinical pregnancy rates. Though, the evidence is poor with a slight increase to high heterogeneity due to the trials on enrolled women offering various kinds of antioxidants. Antioxidants have shown various responses when they are taken alone or in combination exerted a positive effect on pregnancy rate [116]. Moreover, dietary/injectable source of antioxidants during periparturient period provide beneficial effects on pregnancy outcome and growth performance of suckling kids of goats [117, 118].

There is evidence that increased antioxidant levels confront and scavenge ROS in women who have repeated abortions as a result of ROS overload. Previous research has found that women with recurrent abortion have higher levels of lipoperoxides and lower amounts of vitamin A, E, and beta carotene, suggesting the role of ROS. When compared to healthy subject, glutathione activity was low in women who had recurrent abortions [44, 119]. Moreover, selenium concentration from hair samples was also significantly reduced in recurrent abortion than the healthy pregnancies [120]. Increased glucose levels during pregnancy cause teratogenic consequences due to chemical changes and DNA rearrangements. Increased glucose causes the formation of glycation products, which affect genomic function and negatively regulate embryonic development. In diabetic pregnancy, changes in membrane lipids induce biological prostaglandin events, and an enhanced level of ROS causes dysmorphogenesis in the fetus [121]. A reduced level of lipid peroxidation in women with GDM was reported due to depletion of antioxidants activity. Hydroperoxide production affects prostaglandin synthesis patterns, which may result in morbidity owing to antioxidant depletion [122]. GDM also triggers oxidative stress in fetus, thus the intake of antioxidants during pregnancy is essential factor for improving pregnancy health [123]. Further, a detailed description on the role of antioxidants in pregnancy is well-discussed in the previous studies [2, 44, 124–129].

## 8. Conclusion

Antioxidant defense has been established to regulate the generation of ROS; however the increased amount of ROS cannot be controlled, resulting in oxidative stress. So, the potential strategies of antioxidant to decrease ROS levels are critical. According to a large number of studies, oxidative stress is the primary contributing factor in a variety of pregnancy complications. Overstimulation of ROS can cause hyperglycemia, IUGR, miscarriage, and spontaneous abortion throughout all stages of pregnancy. Placental oxidative stress is caused by a number of variables, including maternal history, genetics, and environmental factors, and can lead to negative pregnancy outcomes. Future research should focus on improving the breakdown of intracellular ROS and enhancing antioxidant bioavailability. Targeting signaling molecules with natural bioactive compounds will be used to minimize the occurrence of reproductive problems.

## Figures and Tables

**Table 1:** Positive effect of ROS and antioxidant system in various events of female reproduction and pregnancy outcomes.

Oxidant/antioxidant compounds	Functional activity	Species	References
↑ expression of GSTm2	Preparation of uterus for blastocyst implantation	Mouse	[130]
**↑** GPX and GSR activities	Regulator of H_2_0_2_ and cell death in placental progression	Sheep	[131]
Silence the expression of GPX4	Influencing embryonic brain and heart functions	Mouse	[132]
↓ hydrogen peroxide and superoxide radical	Control uterine contractions	Humans	[133]
↑ SOD1, GPX and GST activities in early pregnancy	Rescue Corpus luteum form apoptosis	Sheep	[134]
↑ CAT and GPX and oviduct GSH in estrus cycle	Govern hydrogen peroxide during fertilization	Cow	[135]
↑ expression of SOD1 in early pregnancy	Directions of luteal functions	Human	[136]
↑ CAT and GPX, and GSH in placenta tissues	Regulates hydrogen peroxide and activation of placental differentiation	Human	[137]
↑ CAT, SOD and GPX in placental and fetal tissues	Defense against ROS toxicity in feto-placental system	Human	[138]
↑ uterine peroxide at blastocyst attachment	Defense to negative effects of hydrogen peroxide actions	Rat	[139]
